# Spatiotemporal Patterns in Production and Consumption of Major Foods in Qinghai, China

**DOI:** 10.3390/foods14050736

**Published:** 2025-02-21

**Authors:** Yexuan Liu, Lin Zhen, Quanqin Shao, Junzhi Ye, Siliang Xie

**Affiliations:** 1 School of Land Science and Space Planning, Hebei GEO University, Shijiazhuang 050031, China; liuyexuan0920@igsnrr.ac.cn; 2 Institute of Geographic Sciences and Natural Resources Research, Chinese Academy of Sciences, Beijing 100101, China; 3 School of Resource and Environment, University of Chinese Academy of Sciences, Beijing 100049, China; 4 School of Ecology, Hainan University, Haikou 570228, China; 5 School of Earth and Ocean Science, University of Victoria, Victoria, BC V8P 5C2, Canada

**Keywords:** production and consumption, supply and demand, self-sufficiency, grain, meat, Qinghai

## Abstract

Food security is an important foundation of national security. Since China entered a new era in 2012, the supply of agricultural and animal husbandry products in Qinghai has continuously enhanced. However, the implementation of ecological policies such as Grain for Green and Grassland Ecological Compensation restricted the cultivation and grazing areas. At the same time, with the improvement in living standards and food consumption demand of local residents, the contradiction between human beings and land has become increasingly prominent. It is necessary to analyze the balance between food supply and demand to evaluate food security. This study used supply–demand analysis and spatial autocorrelation analysis based on county-level statistical data on production and consumption collected through random sampling surveys to reveal the characteristics of the production and consumption of the main food types in Qinghai during 2012–2022 as well as to analyze the food self-sufficiency changes and their spatial clustering features. The results showed that the regions with higher grain and meat production in Qinghai were concentrated in the northeast in the past decade, while the regions with higher consumption were mainly in the counties with larger populations. At the county level, grain could not achieve self-sufficiency, except in northeastern Qinghai; meat was self-sufficient in most counties. Through regional allocation, Qinghai had achieved grain and meat self-sufficiency at the provincial level. The self-sufficiency of grain and meat showed obvious clustering, with high-value clusters of grain self-sufficiency and low-value clusters of meat both distributed in the provincial capital and surrounding areas, which were related to the adjustment of urban residents’ dietary structure from staple foods to diversified foods. This study provides a scientific basis for decision makers when adjusting the agricultural and animal husbandry structure as well as the dietary structure of residents to ensure food security and the sustainable utilization of land resources.

## 1. Introduction

More than halfway through the implementation of the 2030 Agenda for Sustainable Development, the progress toward the goals related to food and agriculture has stagnated or even regressed, making it more challenging to end poverty and hunger as well as improve health and nutrition [1]. The Global Food Security Index (GFSI) has declined for three consecutive years after reaching its peak in 2019 [2] due to the recent impacts of economic crises, regional conflicts, and the COVID-19 pandemic [3,4,5]. During this deteriorating global food environment, the GFSI of China increased by 13.7 during 2012–2022. Among its four key indicators, China scored well on affordability (86.4), availability (79.2), and quality and safety (72.0) [2]. This is inseparable from the efforts China has made in food security since it entered the new era in 2012 [6]. The per capita grain amount was close to 500 kg/person in China, exceeding the international grain security line of 400 kg/person. However, it should also be noted that China has scarce arable land and insufficient water resources [7], accounting for only 8.62% and 5.19% of the Earth, respectively, while the population that needs to be fed accounts for 19.14% of the world population. The GFSI of China also confirmed that due to the inadequate management of water resources, the measure of agricultural water risk had the lowest score, at 25.0, on the indicators of sustainability and adaptation [2]. This means that China’s grain supply and demand will remain in a tight balance for the long term, especially in the plateau of western China where it is difficult to drill wells for water.

Qinghai is located in the Qinghai–Tibet Plateau and the Three-River-Source region, where the food insecurity situation is relatively severe. The local agricultural and animal husbandry production has been facing problems such as low temperature, drought, and land degradation, which have exerted great pressure on the arable land and grassland in ensuring food supply [8,9]. Although China’s grassland ecological compensation policy implemented since 2011 has effectively improved grassland quality [10], measures such as grazing prohibition and the grass–livestock balance have also limited the grazing area and livestock breeding quantity. Therefore, Qinghai has needed to balance the contradiction between ecological environment protection and the development of agriculture and animal husbandry. In addition, urbanization, continuous population growth, and the increasing demand for diversified food by residents have exacerbated the imbalance between food supply and demand in the province [11]. Previous studies on food security have mostly focused on the protection of farmland and crop planting in agricultural areas. This is because agricultural areas are important for ensuring food security; they provide humans with a rich variety of staple food and non-staple food through large-scale planting as well as mature production and processing systems. However, they relatively ignored the role of pastoral areas in the food system. As one of the five major pastoral areas in China, Qinghai’s livestock products such as meat and dairy are important sources of food for local residents [12], and reducing greenhouse gas emissions from animal husbandry is key to achieving a sustainable food system [13]. Integrating grasslands into local production systems and land use decisions could greatly enhance their potential to contribute to food security and sustainable livelihoods.

Ensuring the stability of the food supply chain is crucial for food security. The food supply chain usually consists of five stages: production, processing, wholesale, retail, and consumers [14]. We studied the initial and final stages. Food production was the primary focus of research related to food security, but data were scarce on food production at the international level [15]. Some scholars have used sub-national level data and global agricultural census samples to examine crop production changes at different farm scales [16]. In China, the construction of high-standard farmland was an important measure used to improve grain production capacity [17]. In terms of food consumption, a study predicted that the total global demand for food will increase by 35% to 56% during 2010–2050 [18]. Food demand growth driven by per capita income growth is likely to be more important than growth driven by population growth [19]. As the diet shifts from staple foods to animal-based products, fruits, and vegetables, the demand for land resources increases. For example, the demand for livestock products from Chinese residents has increased by 16% to 30%, which means that China will need 300 × 10^4^–1200 × 10^4^ ha of additional pasture in the period 2020–2050 [20]. “Sustainable intensification” was seen as the preferred approach for addressing this issue. However, intensive production might not be sufficient to meet future food demand without increasing the total environmental burden caused by food production [21]. In addition, many studies have shown that agricultural policy reforms, rural infrastructure construction, and the market monitoring of agricultural and animal husbandry products have provided experience with food security and sustainable production. Firstly, to improve farmers’ production enthusiasm and ensure food supply, China shifted its agricultural and food policy from taxing to subsidizing and protecting agriculture in the mid-2000s [22]. Secondly, the well-developed infrastructure in rural areas was a driving force for the optimal utilization of the available agricultural resources, especially irrigation systems and water management technologies [23]. Thirdly, market monitoring enabled the prompt implementation of measures to regulate food security in the event of significant dynamic changes in the main factors affecting production and consumption [24]. On the whole, to achieve sustainable production and consumption, it is necessary to build a sustainable food security system that can cope with climate change and adapt to future needs; for this purpose, all stakeholders in the food supply chain need to collaborate with each other [25].

In summary, most of the existing studies on food security have focused on analyzing its changes and influencing factors from the perspective of the supply side or demand side in traditional agricultural areas, while there have been few comprehensive analyses on both the supply and demand sides in pastoral areas. The data scales of these studies were not detailed enough, being mostly on the national and regional scales. Given that grain and meat are important components of the diet of residents in Qinghai, this study collected the production and consumption data for grain and meat in 44 county-level areas from 2012 to 2022, quantitatively analyzed the spatial variations and temporal trends in the food supply and demand, and revealed the local food self-sufficiency capacity and its spatial clustering. The research hypotheses included the following: (1) During the study period, with the adjustment of the dietary structure, grain production and consumption in Qinghai have decreased. (2) With the improvement in residents’ living standards, the production and consumption of meat have increased. (3) With the improvement in agricultural and animal husbandry conditions, the local food self-sufficiency has been able to meet the consumption demand of residents. This study provides the basis for further evaluation of food security in Qinghai and provides a decision-making basis for the spatial allocation of grain and meat in pastoral areas.

## 2. Materials and Methods

### 2.1. Study Area

Qinghai province is the intersection of the three major natural regions in China, namely, the eastern monsoon region, the northwest arid region, and the Qinghai–Tibet Plateau region. Specifically, the northeastern part of Qinghai is an agricultural area, involving the Hehuang Valley and Qinghai Lake Basin. This region has relatively concentrated cultivated land and unique grain crops, mainly consisting of cool-loving varieties such as spring wheat, highland barley, potatoes, oats, and peas. The northwest of Qinghai is the Qaidam Basin, which has a dry climate and a combination of agriculture and animal husbandry. The southern plateau of Qinghai is one of the five major pastoral areas and an important livestock production base in China. Alpine grassland and alpine meadow are the main natural grasslands in the southern plateau. The main livestock breeds are yaks, Tibetan sheep, and Hequ horses; the livestock products mainly include wool, cashmere, dairy products, beef, and mutton.

**Figure 1:**
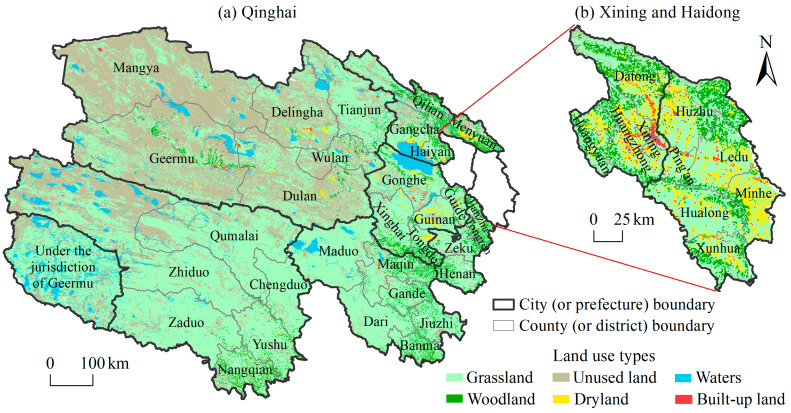
Administrative divisions and land cover of Qinghai province.

Qinghai has jurisdiction over 8 city-level administrative divisions (2 prefecture-level cities and 6 ethnic autonomous prefectures) and 44 county-level administrative divisions (7 municipal districts, 5 county-level cities, 25 counties, and 7 ethnic autonomous counties) (Figure 1), with a total area of 72 × 10^4^ km^2^. The capital of Qinghai is Xining. In 2022, the population of Qinghai was 5.94 million, with a population density of only 8 people/km^2^, and the population distribution was characterized by dense in the east and sparse in the west. The primary industry in Qinghai was dominated by animal husbandry, with the grassland area accounting for 56.29% of the total area in 2020. The cropland has continued to decline in recent years, accounting for 1.24%.

### 2.2. Data Collection

#### 2.2.1. Statistical Survey Data

Two main foods of Qinghai residents were selected for study, namely, grain and meat. Grains (referring to food crops in this study) included cereals (wheat, rice, corn, and others), tubers (sweet potato, potato, and others), and legumes (soybeans and others). Meat products included pork, beef, mutton, poultry, and others. The annual data on grain and meat production, grain and meat consumption per capita, and permanent population at county level from 2012 to 2022 were obtained from yearbooks and bulletins, including Qinghai Statistical Yearbook 2013–2023, China Statistical Yearbook (County-Level) 2014–2023, China’s Ethnic Statistical Yearbook 2016–2022, Xining Statistical Yearbook 2016, and Statistical Bulletin of National Economic and Social Development at the county level during the study period.

Because the statistical caliber of statistical data might differ among years, such as the per capita food consumption data obtained through sampling surveys, the sample sizes and survey subjects varied slightly among the years. This affected the coherence and comparability of the data in the time series to some extent. To reduce the impact of the differences in statistical caliber on the result analysis, this study collected relevant data sources as much as possible and conducted the cross-validation of multiple data sources. This study replaced anomalous values and missing values with the mean of the two adjacent years of data.

#### 2.2.2. Spatial Data

The remote sensing monitoring data of land cover in 2020 were obtained from the Resource and Environment Science and Data Center (https://www.resdc.cn/DOI/DOI.aspx?DOIID=54, accessed on 15 November 2024). They were based on Landsat TM images and were generated by manual visual interpretation. The land cover of Qinghai included six types of grassland, unused land, waters, woodland, dryland, and built-up land; their proportions of area were 56.29%, 33.50%, 4.70%, 4.04%, 1.24%, and 0.23%, respectively. Nearly 90% of the area was composed of grassland (low-cover grassland, mid-cover grassland, high-cover grassland, and others) and unused land (bare rock, sandy land, Gobi desert, and others).

### 2.3. Analysis of Production and Consumption of Grain and Meat

The food self-sufficiency rate is an important measure of food security. It can directly reflect the balance between food production and consumption in a certain period and region. The self-sufficiency rate was calculated as follows:

(1) $${SR}_{in}=\frac{{TFP}_{in}}{{TFC}_{in}}\times 100\%$$

(2) $$T{FC}_{in}={FC}_{in}\times {PRP}_{n}$$

where *SR* represents the self-sufficiency rate in a county, *i* represents the type of food (grain or meat), *n* represents a certain year, *TFP* represents the total production of grain (or meat), *TFC* represents the total consumption of grain (or meat) in a county, *FC* represents grain (or meat) consumption per capita in a county, and *PRP* represents the permanent resident population in a county. If *SR_in_* > 100%, it means that production exceeds consumption, and food self-sufficiency is achieved; if *SR_in_* < 100%, it means a shortage of production, and food self-sufficiency is not achieved; if *SR_in_* = 100%, it means that production is just enough to meet consumption needs.

The gap between food consumption and production was calculated as follows:

(3) $${Gap}_{in}={TFC}_{in}-{TFP}_{in}$$

where *Gap* represents the demand–supply gap of grain (or meat) in a county. If *Gap_in_* > 0, it means the existence of a demand–supply gap, and self-sufficiency is not achieved; if *Gap_in_* < 0, it means that supply exceeds demand, and self-sufficiency is achieved; if *Gap_in_* = 0, it means supply is just keeping up with demand.

### 2.4. Spatial Autocorrelation Analysis of Self-Sufficiency of Grain and Meat

#### 2.4.1. Global Spatial Autocorrelation Analysis

Global spatial autocorrelation analysis was used to reveal whether there was spatial aggregation or dispersion of the self-sufficiency rates of grain and meat in the whole province, as well as the degree of aggregation or dispersion. This study used ArcGIS 10.8 to calculate the Global Moran’s Index of the self-sufficiency rate. The index was calculated as follows:

(4) $$Global\;Moran^{’}s\;I=n\frac{\sum_{i=1}^{n}\sum_{j=1}^{n}w_{ij}(x_{i}-\bar{x})(x_{j}-\bar{x})}{(\sum_{i=1}^{n}\sum_{j=1}^{n}w_{ij})\sum_{i=1}^{n}{\left(x_{i}-\bar{x}\right)}^{2}}$$

where *n* represents the total number of county-level administrative divisions in Qinghai (*n* = 44); $x_{i}$ and $x_{j}$ represent the self-sufficiency rate of the *i*-th county and *j*-th county, respectively; and $w_{ij}$ represents the spatial weight matrix.

The calculation of Moran’s I requires at least 30 samples. This study had 44 county-level samples used for calculation, which met the requirement for sample size. The significance level of Moran’s I was tested using the standard *z*-score. If *p* < 0.1 and *z* > 1.65 or <−1.65, it indicated that the self-sufficiency rate was not randomly distributed. The Global Moran’s I ranges from [−1,1]. If the index value was close to ±1, it meant that there was a significant spatial autocorrelation of the self-sufficiency rate; if the index was close to 0, it meant that there was no significant spatial autocorrelation. A positive index indicated that similar self-sufficiency rates were clustered spatially, and a negative index indicated the existence of spatial heterogeneity in the self-sufficiency rates.

#### 2.4.2. Local Spatial Autocorrelation Analysis

Furthermore, the local spatial autocorrelation was used to determine where the self-sufficiency rates were clustered or dispersed. The index was calculated as follows:

(5) $$Local\;Moran^{’}s\;I=\frac{\sum_{j=1}^{n}w_{ij}(x_{i}-\bar{x})(x_{j}-\bar{x})}{\sqrt{\sum_{i=1}^{n}\frac{\left(x_{i}-\bar{x}\right)}{\left(n-1\right)}}}$$

A positive Local Moran’s I indicated that the self-sufficiency rate in the *i*-th county was similar to that of its neighboring counties; that is, high values were surrounded by high values, or low values were surrounded by low values. A negative index indicated that the self-sufficiency rate in the *i*-th county was different from that of its neighbors; that is, high values were surrounded by low values, or low values were surrounded by high values.

This study used GeoDa 1.22 to calculate the Local Moran’s I of the self-sufficiency rates of grain and meat, and this study plotted a Moran scatter plot and the local indicators of spatial association (LISA) cluster map. In the Moran scatter plot, the four quadrants represent different types of spatial correlation: the first and third quadrants represent positive correlation types, while the second and fourth quadrants represent negative correlation types. The points in the first to fourth quadrants belong to four types: high–high cluster, low–high outlier, low–low cluster, and high–low outlier.

## 3. Results

### 3.1. The Spatiotemporal Variation in Grain and Meat Production

**Figure 2:**
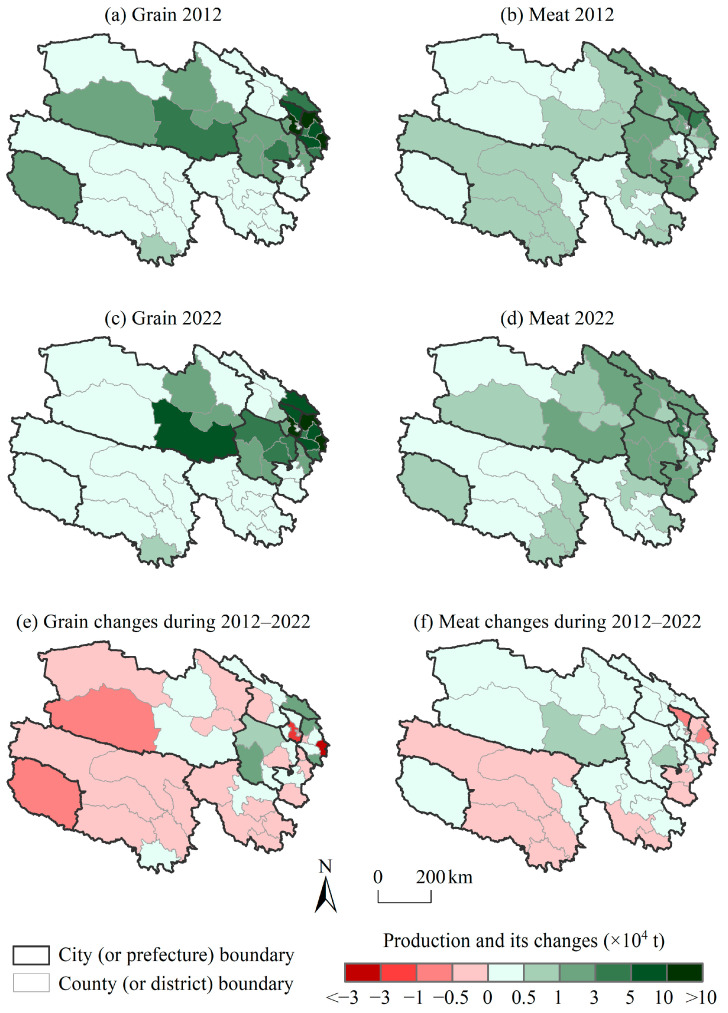
The distribution of and changes in grain and meat production at the county level in Qinghai from 2012 to 2022.

The areas with high grain production in Qinghai were concentrated in the Yellow River–Huangshui River Valley and the southeast of the Qaidam Basin (Figure 2). In 2012, the grain production of Minhe, Huzhu, Huangzhong, and Datong accounted for 50.14% (54.87 × 10^4^ t) of the total production in the whole province, with all four counties producing more than 8.00 × 10^4^ t. The terrain in these areas is relatively flat, and there are more abundant arable land and irrigation water resources, so they are the traditional grain-production areas. However, in the southern plateau of Qinghai, the climate is cold, and the crop-growing season is short. In addition, the lack of arable land has limited the scale of grain production in the southern plateau, resulting in low grain output. From 2012 to 2022, grain production in Qinghai showed a trend of first increasing and then decreasing, increasing from 109.44 × 10^4^ t in 2012 to 188.47 × 10^4^ t in 2017 and then decreasing to 107.27 × 10^4^ t in 2022. In terms of spatial changes, grain production in the major grain-producing areas in northeastern Qinghai increased, with the grain production growth in Menyuan, Xinghai, Huzhu, and Xunhua all exceeding 1.00 × 10^4^ t. The grain production in non-major grain-producing areas generally declined, such as in the southern plateau and the western Qaidam Basin.

The areas with high meat production included the Yellow River–Huangshui River Valley, the Qilian Mountains, and the southern plateau in Qinghai (Figure 2). The overall output structure was beef > mutton > pork > poultry. From 2012 to 2022, the total meat production in Qinghai fluctuated and increased (36.96 × 10^4^ t~44.88 × 10^4^ t), with the output exceeding 3.00 × 10^4^ t in Datong, Huzhu, Huangzhong, and Ledu. The meat production increased in most counties, such as in Guinan, Dulan, Jiuzhi, Menyuan, and Huangzhong, where the production increased by more than 0.30 × 10^4^ t. This could not have been achieved without the implementation of breeding subsidy policies and the promotion of advanced breeding technologies, which not only expanded the scale of breeding but also increased the meat production per unit of livestock. The meat production in the Three-River-Source region of southwestern Qinghai slightly decreased during 2012–2022, which was related to the restriction on the livestock number raised due to measures regarding grazing prohibition and the grass–livestock balance.

### 3.2. The Spatiotemporal Variation in Grain and Meat Consumption

**Figure 3:**
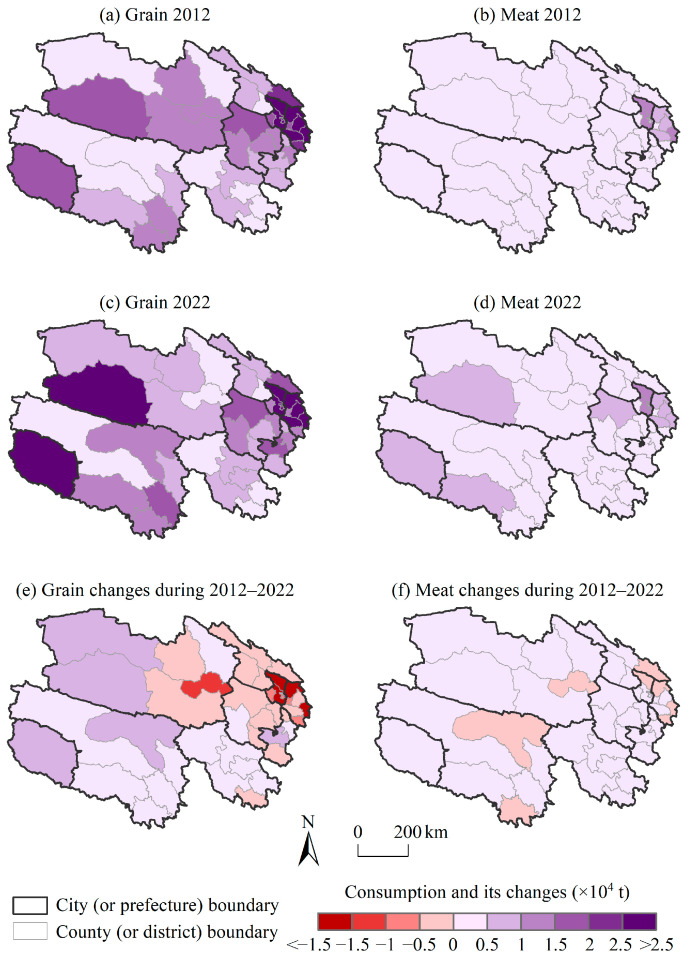
The distribution of and changes in grain and meat consumption at the county level in Qinghai from 2012 to 2022.

The areas with high grain consumption in Qinghai were cities and agricultural counties with relatively large populations and developed economies, such as Xining, Delingha, and Geermu (Figure 3). The total grain consumption in these three cities was 30.34 × 10^4^ t and 27.70 × 10^4^ t in 2012 and 2022, respectively, accounting for 38.70% and 40.02% of the whole province. The residents of pastoral counties had lower demand for grain; for example, the grain consumption in Zhiduo was only 0.50 × 10^4^ t in 2022. The spatial differences in meat consumption in Qinghai were not very obvious, with slightly higher consumption in the provincial capital Xining and its surrounding areas.

From 2012 to 2022, with the improvement in residents’ living standards in Qinghai, their food demand gradually diversified. It was manifested as a continuous decline in grain consumption in the whole province from 78.39 × 10^4^ t to 69.21 × 10^4^ t and a continuous increase in meat consumption from 14.54 × 10^4^ t to 19.79 × 10^4^ t. The grain demand in Huangzhong decreased the most (2.31 × 10^4^ t). Only seven counties in the province showed a small decrease in meat consumption (≤0.20 × 10^4^ t), including Wulan, Nangqian, Minhe, Datong, Huzhu, Xunhua, and Qumalai (Figure 3). It could be seen that the food consumption structure of the residents in Qinghai was upgrading, which was similar to the transformation of the national food consumption structure, shifting from plant-based foods to animal-based foods.

### 3.3. The Spatiotemporal Variation in Self-Sufficiency of Grain and Meat

**Figure 4:**
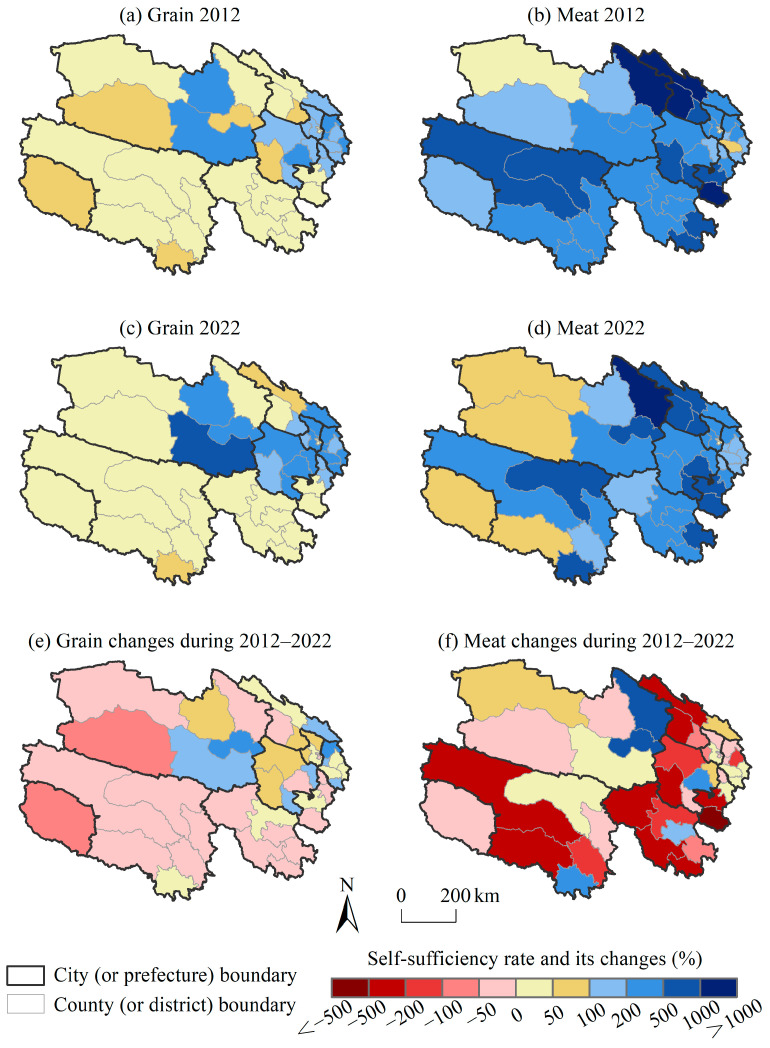
The distribution of and changes in self-sufficiency rate of grain and meat at the county level in Qinghai from 2012 to 2022.

The spatial distribution of grain self-sufficiency varied significantly (Figure 4). The Yellow River–Huangshui River Valley and the southeast of the Qaidam Basin had concentrated arable land and high grain production, achieving a grain supply greater than the household consumption. At the county level, almost all counties under the jurisdiction of Xining, Haidong, and Hainan Tibetan Autonomous Prefecture achieved grain self-sufficiency in 2022, with self-sufficiency rates of over 100%. Delingha, Wulan, and Dulan in Haixi Mongolian and Tibetan Autonomous Prefecture also achieved grain self-sufficiency, with Dulan’s self-sufficiency rate reaching 600%. The high self-sufficiency rates were conducive to reducing the price fluctuations caused by food shortages, and the surplus grain could be used for external sales, which increased local fiscal revenue and farmers’ economic benefits. However, excessive grain surplus might cause storage and sale problems, such as grain spoilage and grain backlog, which also affect the production enthusiasm of farmers. Except for the above areas, the grain self-sufficiency rates in the other areas of Qinghai were less than 100%, especially in the southern plateau. This indicated that the supply of grain failed to meet the consumption demands of local residents. Overall, the grain self-sufficiency rates decreased in half of the counties (22 counties), while they increased in the other half (22 counties) from 2012 to 2022.

Most counties in Qinghai had good meat self-sufficiency and met the dietary needs of local residents (Figure 4), especially for beef and mutton. There were a few regions with self-sufficiency rates below 100%, such as traditional agricultural areas like Xining, whose animal husbandry production could not meet local consumption demands. For another example, the economies of Geermu and Mangya were dominated by mining and chemical industries, rather than animal husbandry, so they also did not achieve meat self-sufficiency. About two-thirds of the counties (29 counties) experienced a decrease in meat self-sufficiency, while about one-third (15 counties) experienced an increase in the past decade.

**Figure 5:**
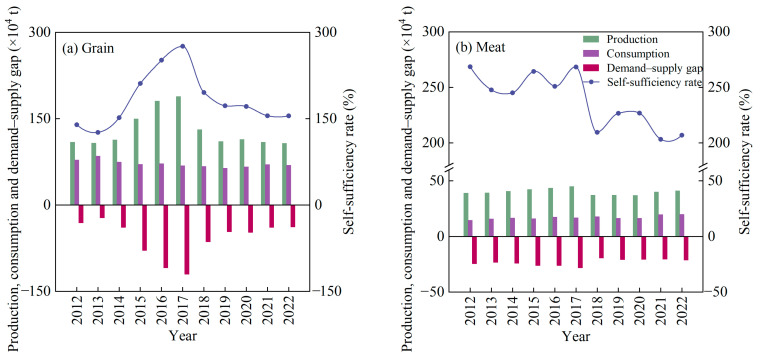
The annual changes in self-sufficiency of grain and meat at the provincial level in Qinghai.

Although not all grain and meat products were self-sufficient at the county level, these foods could be circulated within the province through regional surplus–shortage adjustment to achieve food self-sufficiency and optimal allocation. Therefore, at the provincial level, Qinghai was able to ensure self-sufficiency for both grain and meat in the past decade, and there was no demand–supply gap for these two major types of food (Figure 5). The self-sufficiency rates of grain were between 126% and 276%, showing a trend of first increasing and then decreasing. The main reasons for the decline in the grain self-sufficiency rate since 2017 were reduced supply. The meat self-sufficiency rates were between 203% and 269%, showing a fluctuating downward trend because the growth rate of the demand was faster than that of the supply. Therefore, in the case of maintaining a stable supply of two main foods in Qinghai, in the future, we should focus on coping with the potential mismatch between the supply and demand for various types of foods caused by the changes in residents’ diet structure as well as on the food security risks caused by uneven regional distribution.

### 3.4. Spatial Autocorrelation of Self-Sufficiency of Grain and Meat

#### 3.4.1. Global Spatial Autocorrelation at the Provincial Level

The Global Moran’s I values of grain and meat self-sufficiency were greater than 0, with *z* > 1.65 and *p* < 0.1 (Table 1), passing the significance test. The self-sufficiency rates of grain (or meat) showed positive spatial autocorrelation throughout the whole province. This indicated the existence of the clustered distribution if the self-sufficiency rates. The spatial clustering of meat self-sufficiency was more significant than that of grain. From 2012 to 2022, the Global Moran’s I of self-sufficiency for both grain and meat increased, indicating that similar self-sufficiency rates were becoming more concentrated spatially.

#### 3.4.2. Local Spatial Autocorrelation at the County Level

**Figure 6:**
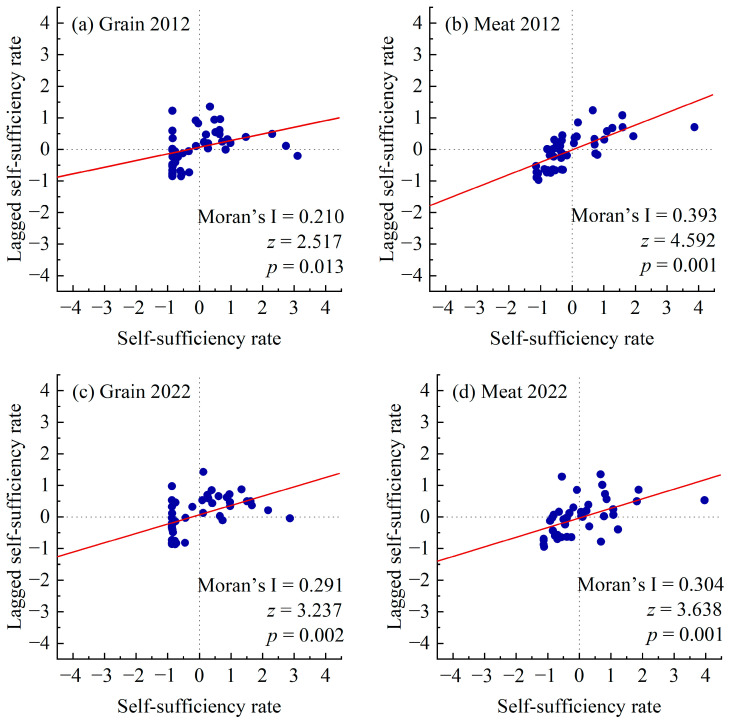
Moran scatter plot of the self-sufficiency for grain and meat at the provincial level in Qinghai.

In the Moran scatter plot, all four Moran’s I are all greater than 0, indicating that the spatial autocorrelation was dominated by positive correlation (Figure 6). That is, counties with high self-sufficiency rates often clustered together, forming hotspot areas; counties with low self-sufficiency were also concentrated, forming coldspot areas. The scatter points were mainly distributed in the first quadrant (high–high cluster) and the third quadrant (low–low cluster), which also proved that the high-value counties or low-value counties regarding self-sufficiency showed clustering characteristics. The food self-sufficiency of a county was influenced by its surrounding counties, and thus they gradually converged. This might have been due to factors such as the flow of agricultural and pastoral resources, technology popularization, and policy transmission caused by geographical proximity, leading to similar self-sufficiency.

**Figure 7:**
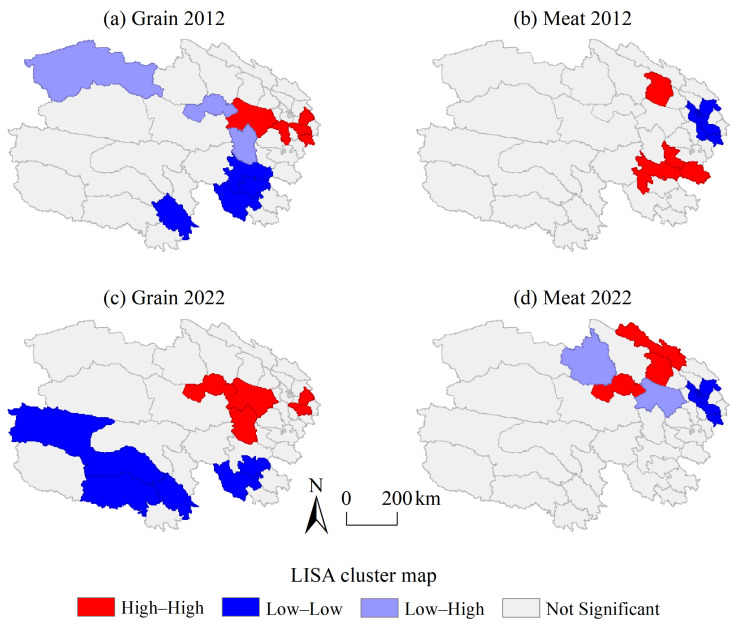
LISA cluster map of the grain and meat self-sufficiency at the county level in Qinghai.

Specifically, areas with high self-sufficiency rates for grain were clustered in the Yellow River–Huangshui River Valley in 2012 (high–high cluster), mainly including Ledu, Hualong, and Gonghe, and then Wulan and Xinghai were added in 2022 (Figure 7). The low self-sufficiency rates for grain were concentrated in the Three-River-Source region (low–low cluster), involving Yushu, Zhiduo, Maqin, Gande, and Dari. The inability to implement large-scale crop cultivation in the Three-River-Source region fundamentally constrained the layout of grain production, making it difficult to achieve a high level of self-sufficiency for grain. While protecting the fragile ecological environment of the Three-River-Source region, appropriate agricultural development models should be explored. These low–low cluster areas could engage in grain trading with high–high cluster areas, thus establishing an inter-regional food mutual assistance mechanism as well as enhancing the capacity and resilience of the food security in the entire region.

The high meat self-sufficiency rates in 2012 were clustered in Maqin, Henan, Tongde, and Gangcha (high–high cluster), and the clustered areas were Gangcha, Qilian, and Wulan in 2022 (Figure 7). Xining and its surrounding counties were clustered areas of low meat self-sufficiency (low–low cluster), including Xining, Huzhu, Huangzhong, Ping’an, Jianzha, Hualong, and Xunhua. With the continuous growth in the population in Xining and its surrounding areas, the meat demand of urban residents was rapidly rising. At the same time, urban expansion led to some land being used for urban construction and industrial development, squeezing the development space for animal husbandry. Xining could consider developing suburban animal husbandry or strengthening cooperation with major meat-producing areas to ensure meat self-sufficiency.

## 4. Discussion

### 4.1. Comparison with Previous Research

Through the analysis of the results, some of the research hypotheses were proven, as follows: (1) Hypothesis 1 was partially supported. The results showed that grain production showed an increasing trend during the first half of this study period rather than the assumed continuous decline during this study period. (2) Hypothesis 2 was confirmed. The results indicated that both production and consumption of meat were continuously increasing with fluctuations, which was consistent with the hypothesis. (3) Hypothesis 3 was partially proven. Although the self-sufficiency of grain and meat met the consumption needs of residents at the provincial level, many counties in the northwest and south were below 100% self-sufficient. So, the assumption that the local food self-sufficiency was able to meet the demands of residents was not always valid in every case.

In terms of food supply and demand, many studies have approached the analysis from the perspective of ecological products and ecosystem services. For example, Huang et al. revealed that the food supply of ecological products in Qinghai from 2000 to 2020 was basically in a state of supply exceeding demand, and areas with high-risk levels of supply and demand were mainly distributed in urban areas characterized by high supply and high demand [26]. These conclusions coincided with our research. They also mentioned that optimizing supply–demand regulation within regions could improve the allocation efficiency of food supply and demand, and we also support this strategy. Ma et al. calculated the ecological security bottom line of supply and demand by combining food demand and ecosystem service values in Qinghai [27]. They found that under the constraint of the ecological red line and permanent basic farmland, the disorderly expansion of construction land has been restrained, which has been crucial for balancing ecological security and food security in Qinghai. Another study on the Gansu province adjacent to southeastern Qinghai showed that the food supply of the ecosystem services nearly doubled from 2002 to 2018, while the demand of residents for high-energy food, such as wheat, decreased [28]. Our result is generally consistent with this; that is, grain production showed an increasing trend, while grain consumption decreased before 2018. However, some scholars have pointed out that the demand of residents for grain would increase [29]. This is because residents’ demand for food in northwest China has gradually shifted from rations to animal products, which require more raw grains under the same calorie supply conditions.

In terms of food self-sufficiency, Shi et al. calculated that the self-sufficiency rate of grain in the Qinghai–Tibet Plateau reached 173.03% in the period between 2010 and 2016, but there remained serious spatial imbalances [30]. This is consistent with the grain self-sufficiency rate of 180.75% in 2016 in our study, which is of the same magnitude and close in value. Another study also calculated a grain self-sufficiency rate of 120.21% for the Qinghai–Tibet Plateau in 2020, which is also close to the 113.88% in our study [31]. These comparisons to some extent demonstrate the credibility of our research results. Wang et al.’s research showed that, in the Qinghai–Tibet Plateau as a whole, food consumption was guaranteed at the well-off level from the perspective of nutritional demand during 2010–2019, and the conflict between food security and ecosystem conservation could be managed without sacrificing nature [32]. This is similar to our result of achieving the self-sufficiency of major foods in Qinghai as a whole.

### 4.2. Prospects for Future Research on Factors Influencing Food Security

The production of agriculture and animal husbandry, as well as the capacity for food self-sufficiency, are comprehensively influenced by multiple factors. The impacts of climate change on agriculture and animal husbandry are long-term and complex, such as the effects of temperature and precipitation changes on crop growth cycles and livestock breeding [33,34]. Agricultural production in Qinghai is at high risk of being affected by low temperature and drought. Increasing the construction of irrigation infrastructure and utilizing intelligent facilities to reduce climate disaster risks urgently need to be improved in Qinghai. Establishing a predictive model to conduct correlation analysis between long-term meteorological data and production data and revealing how climate changes affect grain and meat production in different regions of Qinghai can provide a scientific basis for regional agricultural and animal husbandry production to cope with climate change.

The impact of urbanization and population mobility on food production and self-sufficiency in underdeveloped areas has gradually emerged in recent years [35]. The migration of the population to cities has led to a decrease in the labor force in rural and pastoral areas, affecting the scale and mode of agricultural and pastoral production [36]. At the same time, urbanization has also changed the distribution of the consumer market, food consumption concepts, and demand characteristics [37]. It is necessary to analyze the demand changes for food quality, variety, and safety, as well as how these changes feed back into the production process. This provides guidance for agriculture and animal husbandry production to adapt to market demand, so as to ensure food self-sufficiency under the background of urbanization and the non-agriculturalization of the rural labor force.

Agricultural and animal husbandry production is also influenced by multiple policies, such as how to achieve synergy between ecological protection policies and agricultural as well as animal husbandry development policies [38,39]. It is necessary to analyze the effects of these policies in different regions and production processes, comprehensively evaluate the long-term effects and comprehensive impacts of existing policies, and explore paths to optimize policy combinations. In addition, it is also very meaningful to determine the changes in the number of livestock raised due to ecological protection policies. It is possible to model a forecast for the scenario of a reduction in the number of livestock, which leads to a decrease in meat production, and for the possible long-term consequences of this trend for food security.

## 5. Conclusions

Exploring the characteristics and relationships between food production and consumption in pastoral areas helped to understand the food security situation of local residents. This study quantitatively evaluated the production, consumption, and self-sufficiency of major food types at the county and provincial levels in Qinghai during 2012–2022. The main conclusions are as follows:

During the study period, total grain production in Qinghai first increased and then decreased, while the consumption showed a downward trend. The areas with higher grain production were mainly concentrated in the agricultural counties in the Yellow River–Huangshui River Valley and the southeast of the Qaidam Basin, while the production in the non-major grain-producing areas in the west and south Qinghai were lower and continued to decline. The areas with high grain consumption were mainly located in populous cities, such as Xining and Haidong, and their surrounding agricultural counties. Meat production and consumption both continuously increased, and high-value areas for meat production and consumption were also distributed in urban and surrounding agricultural areas rather than pastoral areas. In the past ten years, the dietary structure of the residents especially in the northeastern agricultural areas improved. The proportion of grain in the residents’ diet decreased and meat consumption increased in most counties, but meat production did not grow as fast as its consumption.

Qinghai was able to ensure the overall self-sufficiency of grain and meat at the provincial level, with grain self-sufficiency rates ranging from 126% to 276% and meat self-sufficiency ranging from 203% to 269%. Since 2017, the self-sufficiency rates of grain and meat significantly decreased in the province as a whole. The spatial variations in self-sufficiency at the county level were great. The self-sufficiency rates of agricultural counties in the northeast Qinghai were relatively good, which could meet residents’ demands. However, the grain production in the industrial counties in the northwest and the pastoral counties in the south were insufficient, resulting in a lower level of self-sufficiency. Therefore, it is necessary to strengthen regional regulation and management of local major foods, thereby achieving the rational flow and optimized allocation of food resources and improving the resource utilization efficiency of the entire region.

## Figures and Tables

**Table 1:** The global autocorrelation analysis of self-sufficiency of grain and meat.

Year	Food Type	Moran’s I	*z*-Score	*p*-Value
2012	Grain	0.098	1.949	0.051 *
2022	Grain	0.109	2.099	0.036 **
2012	Meat	0.304	5.346	0.001 ***
2022	Meat	0.311	5.482	0.001 ***

Note: * represents *p* < 0.1, ** represents *p* < 0.05, *** represents *p* < 0.01.

## Data Availability

The original contributions presented in this study are included in the article. Further inquiries can be directed to the corresponding author.
